# Impact of establishing a respiratory high dependency unit for SCAP patients on the therapeutic effect, prognosis, and expenditure: a retrospective case–control study

**DOI:** 10.1038/s41598-022-14705-w

**Published:** 2022-06-23

**Authors:** Qin Yalan, Tong Jin

**Affiliations:** 1 Department of Critical Care Medicine, The Second Affiliated Hospital of Chongqing Medical University, Chongqing, 400010 China; 2 Department of Respiratory Medicine, The Second Affiliated Hospital of Chongqing Medical University, 76# Linjiang Road, Yuzhong district, Chongqing, 400010 China

**Keywords:** Medical research, Outcomes research, Disease-free survival, Respiratory distress syndrome

## Abstract

To explore the effects of establishing a high dependency unit (HDU) on the prognosis, outcome, and expenditure of patients with severe community-acquired pneumonia (SCAP). 108 SCAP patients were recruited from the respiratory intensive care unit (RICU) of the Second Affiliated Hospital of Chongqing Medical University, Chongqing, China. Of these, 87 qualified the study-selection criteria and were divided into HDU group (treated in HDU after discharge from RICU prior to transfer to normal unit) (n = 40) and normal group (not treated in the HDU) (n = 47). In the 87 patients, 40 were divided into HDU group, which meant they transferring to HDU when got stable while another 47 were divided into normal group which meant they staying longer in RICU and transferring to normal unit when got stable. Acute Physiology and Chronic Health Evaluation II (APACHE II) and Sequential Organ Failure Assessment (SOFA) scores, duration of mechanical ventilation, hospital infection, intensive care unit syndrome, length of stay, and expenditure were compared between the two groups. The primary outcome was discharging from hospital while the secondary outcome was length of stay. There was no significant difference with respect to noninvasive ventilation time, oxygenation index, or APACHE II and SOFA scores at admission or discharge from RICU (P > 0.05). The mean invasive ventilation time (176 ± 160 h) of the HDU group was not significantly different from that in the normal group (206 ± 179 h). The period of sequential noninvasive ventilation in the HDU group (135 ± 82 h) was significantly shorter than that in the normal group (274 ± 182 h, P < 0.05). The HDU group had a shorter length of stay in hospital and RICU, and incurred lesser expenditure than patients in the normal group (P < 0.05). Patients in HDU group had almost the same therapeutic effect with shorter length of stay in hospital and RICU, and lesser expenditure.

## Introduction

Globally, severe community-acquired pneumonia (SCAP) is a leading cause of sepsis in hospitalized patients and death from infectious diseases^1^. The mortality rate of SCAP patients who are admitted to intensive care unit (ICU) is approximately 30%^2^. Indeed, SCAP is associated with a high clinical burden besides the economic aspects. In a study by Dupuis et al., the median ICU stay and length of hospital stay of SCAP patients were 8 (4, 16) and 17 (10, 29) days, respectively, with the mean cost of €19,008 ± €17,218^3^. In the study by Çelikhisar et al., the length of stay in ICU of some of the SCAP patients was 19.05 ± 9.48 days^4^. Thus, improving the management of these patients is a key imperative.

Traditionally, SCAP patients are first admitted to the respiratory intensive care unit (RICU) and are shifted to normal units (NU) when their condition becomes stable; this is referred to as the RICU-NU pattern. However, prolonged treatment in RICU may cause delirium, secondary infection, and reduction in bed rotation rate^5^. In order to address these problems and simultaneously deliver enhanced respiratory care outside of the intensive care setting, a dedicated respiratory high-dependency unit (HDU), led by the same respiratory physicians and supported by the same specialist respiratory nursing and physiotherapy staff, was established beside the RICU. Respiratory support here is defined as noninvasive ventilation (NIV) and high-flow nasal oxygen (HFNO)^6^. SCAP patients are first admitted to RICU and then transferred to HDU, followed by their transfer to normal units when their condition improves. We called this as RICU-HDU-NU pattern, in which there is a transitional phase of treatment in the HDU. This pattern of care may reduce the length of stay (LOS) in the RICU, expenditure, and the incidence of ICU syndrome.

Some countries have developed HDUs after the breakout of COVID-19^7^. Nevertheless, most of these HDUs were used as a substitute for ICU rather than as a transitional unit. Even some were just used for speeding up the bed turnover and ease the burden of RICU beds^8^. In our hospital, we want to establish HDUs to transform the pattern of care of SCAP patients resulting in medical and economic value.

In this study, we compared the RICU-NU pattern and RICU-HDU-NU pattern to explore the effects of establishing a HDU on the prognosis, length of stay, and expenditure of SCAP patients.

## Methods

### Study design and patient recruitment

This was a single-center, retrospective, case–control study. All patients with SCAP who were admitted to the RICU of The Second Affiliated Hospital of Chongqing Medical University, Chongqing, China between 1 March 2020 and 31 May 2021 were identified from the database. This database prospectively records the detailed clinical data including the history of present illness, past medical history, and personal history. Among these patients, some patients were transferred to HDU after extubation or when invasive mechanical ventilation (IMV) was deemed unnecessary and transferred to normal units later upon stabilization of their condition. This group of patients was classified as the treatment group (referred to as the HDU group). Other patients were never shifted to HDU and could not be transferred to normal units after extubation until their condition stabilized with nasal tube oxygenation. This group of patients was classified as the control group (referred to as the normal group). The end-point of the observation was discharge of patients.

A total of 87 patients were included in the study, 40 in the HDU group and 47 in normal group.

### Inclusion criteria

1. Age > 18 years;
2. Qualified the diagnostic criteria of SCAP (2007 ATS/IDSA guidelines for the treatment of community-acquired pneumonia)^9^). Main criteria: (a) requirement of IMV; (b) development of shock which required use of vasoactive agents. Secondary criteria: (a) respiratory rate > 30 beats per minute; (b) oxygenation index < 250 mmHg; (c) involvement of multiple lobes; (d) altered consciousness; (e) Blood Urea Nitrogen > 20 mg/dL. (f) White blood cell count < 4 × 10^9^; g) platelet count < 100 × 10^9^; (h) core body temperature < 36 ℃; (i) fall in blood pressure requiring fluid resuscitation. Patients who satisfy one main criteria or 3 secondary criteria can be diagnosed as SCAP.
3. Need for IMV and/or NIV and/or HFNO.
4. Admitted to RICU.
5. Length of hospital stay > 3 days.

### Exclusion criteria

1. Patients who were discharged or transferred to another hospital during treatment.
2. Patients who did not require mechanical ventilation (invasive or noninvasive).
3. Patients who could not be extubated in RICU.

### Ethical standards statement

Our study has been approved by the ethics committee of the second affiliated hospital of Chongqing Medical University and have therefore been performed in accordance with the ethical standards laid down in the 1964 Declaration of Helsinki and its later amendments. And the ethic ID was number 59 in 2015.

### Laboratory tests

Arterial blood gas analysis was performed for all patients at the time of admission to RICU, discharge from RICU, and discharge from the hospital. Blood routine, coagulogram, hepatic and renal function tests were performed at the time of admission to RICU and repeat tests were performed every 3 days. We scored Acute Physiology and Chronic Health Evaluation II (APACHE II) and Sequential Organ Failure Assessment (SOFA) scores at the time of admission to and discharge from the RICU.

### Treatment

Patients in the RICU and HDU were treated by the same medical and nursing team, but with different nurse-to-patient ratio (1:2 vs 1:4, respectively). The treatment strategy was based on the 2007 ATS/IDSA guidelines for the treatment of community-acquired pneumonia. Briefly, the treatment included 3 main aspects: respiratory support, antibiotics, and general supportive measures.

Respiratory support was provided based on a standard protocol. Oxygen was administered via nasal tube to patients with oxygenation index > 250 mmHg. NIV and HFNO therapy were provided to patients with oxygenation index between 100 and 250 mmHg. Endotracheal intubation and invasive mechanical ventilation were considered for patients with oxygenation index < 100 mmHg. In most cases, the final decision was made by the medical team leader based on the specific circumstances. Moxifloxacin, Piperacillin/Tazobactam and Carbapenems were the most commonly used antibiotics for SCAP patients. Sometimes, antifungal drugs and glycopeptides antibiotics were also required. The final choice was made by the medical team leader based on culture results and drug sensitivity test or just experience.

In the HDU group, the endotracheal tube was removed from the intubated patients as soon as possible after they passed the spontaneous breath test aiming for target oxygen saturation (SaO_2_) of 92–96%^10^ and transferred to HDU with the support of NIV or HFNO, if necessary. Other patients who did not require IMV from the very beginning were transferred to HDU 1–2 days after admission to the RICU supported by NIV or HFNO. Subsequently, when their condition stabilized, they were transferred to normal units.

In the normal group, the criteria for removal of the endotracheal tube in intubated patients was the same as that in the HDU group. After extubation, they received NIV or HFNO, if necessary. Other patients in the normal group who did not require IMV from the beginning stayed in the RICU with the support of NIV or HFNO, until oxygen therapy via nasal tube could maintain their oxygenation level.

In brief, according to *Guidelines for construction and management of intensive care medicine (2020)*^11^ released by General Office of the Ministry of Health in China and relevant regulations of our hospital, we set the criteria for transition as following: 1. Transition from RICU to HDU: (a) Patients no longer required IMV but required NIV or HFNO to maintain oxygen saturation more than 93%. (b) Patients had stable hemodynamics and didn’t require vasoactive drugs any more or just very small dose of norepinephrine (less than 0.2ug/kg.min) was needed. (c) The functions of other organs were basically restored. 2. Transition from RICU to NU: (a) Patients no longer required IMV or NIV or HFNO and the nasal tube oxygen could maintain their oxygenation level. (b) The same, they had stable circulation and recuperative organ functions. 3. Transition from HDU to NU: Patients no longer required NIV or HFNO.

### Statistical analyses

All analyses were conducted using SPSS 26.0. Normally-distributed continuous variables are expressed as mean ± standard deviation while non-normally continuous variables are expressed as median (interquartile range). Categorical variables are summarized as frequency (percentage). The *t* test or Mann–Whitney U test was used to analyze continuous variables, and the Fisher exact test or Pearson χ^2^ test was used for categorical variables.

### Ethics approval and consent to participate

The experimental protocol was established, according to the ethical guidelines of the Helsinki Declaration and was approved by the Human Ethics Committee of the Second Affiliated Hospital of Chongqing Medical University. Written informed consent was obtained from individual or guardian participants.

### Patient and public involvement statement

Patients or the public WERE NOT involved in the design, or conduct, or reporting, or dissemination plans of our research.

## Results

**Figure 1:**
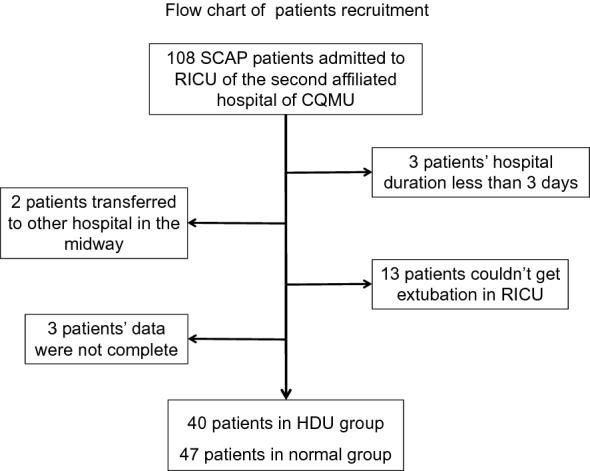
Flow chart of patients recruitment.

During the study reference period, 115 patients were diagnosed with SCAP on admission. Among these, 3 patients were excluded as the length of hospital stay was less than 3 days and 2 patients were excluded as they were transferred to another hospital. Moreover, complete data were not available for 3 patients. Twenty patients could not get extubated in the RICU (Fig. 1). Finally, 87 patients (58 male, 66.7%) were included in this study (47 in the normal group and 40 in the HDU group). The mean age of patients was comparable in the two groups (HDU group: 66.4 ± 20.7 years; normal group: 65.2 ± 18.5 years; P > 0.05). Most patients had comorbid conditions. Hypertension, diabetes mellitus (DM) and coronary heart disease (CHD) were the most common comorbid conditions. Fourteen patients (35.0%) in the HDU group had one of the above diseases compared to 17 patients (36.2%) in the normal group. However, some patients had more than one comorbid conditions. Six patients in the HDU group had two comorbid conditions compared with 8 in the normal group. Four patients in the HDU group had three comorbid conditions compared with 2 in the normal group. In the HDU group, 2 patients had autoimmune disease (AID) and 4 had chronic obstructive pulmonary disease (COPD); the corresponding numbers in the normal group were 6 and 2, respectively. All of these comorbidities were reported to be stable in the chronic phase by the patients’ clinicians (Table 1).

**Table 1:** Comparison of the characteristics of patients in the HDU and normal groups.

	HDU group, n = 40	Normal group, n = 47	t/X^2^/M-W	P
Age	66.4 ± 20.7	65.2 ± 18.5	0.276	0.783
**Sex (n, %)**				
Male	25 (62.5)	33 (70.2)	0.578	0.447
Female	15 (37.5)	14 (29.8)		
**Comorbid conditions**				
One of hypertension, DM and CHD	14 (35.0)	17 (36.2)	0.013	0.910
Two of hypertension, DM and CHD	6 (15.0)	8 (17.0)	0.065	0.798
Hypertension, DM and CHD	4 (10.0)	2 (4.3)	1.111	0.407
AID	2 (5.0)	6 (12.8)	1.561	0.279
COPD	4 (10.0)	2 (4.3)	1.111	0.407
Others	5 (12.5)	8 (17.0)	0.348	0.556
**APACHE II score**				
At Admission to RICU	14.5(8.0,19.0)	15.0 (12.0, 24.3)	706.0	0.063
At discharge from RICU	10.0 (6.3, 13.8)	11.0 (5.0, 17.8)	830.5	0.657
**SOFA score**				
At admission to RICU	5.0 (3.0, 6.0)	5.0 (3.0, 8.0)	788.5	0.252
At discharge from RICU	2.0 (2.0, 4.0)	2.0 (1.0, 5.8)	836.0	0.689
**Oxygenation index**				
At admission to RICU	208.4 ± 86.9	182.0 ± 81.0	1.460	0.148
At discharge from RICU	256.7 ± 81.5	240.3 ± 121.5	0.737	0.463
At discharge from hospital	247.3 ± 81.1	225.9 ± 120.5	0.936	0.352

Data presented as frequency (%), mean ± standard deviation or as median (interquartile range).

*APACHE II* acute physiology, age, chronic health evaluation II, *SOFA* sequential organ failure assessment, *DM* diabetes mellitus, *CHD* coronary heart disease, *AID* autoimmune disease, *COPD* chronic obstructive pulmonary disease, *RICU* respiratory intensive care unit, *HDU* high dependency unit.

There was no significant between-group difference with respect to APACHE II and SOFA scores at the time of admission or discharge from RICU (P > 0.05). Also, there was no significant between-group difference with respect to oxygenation index at the time of admission or discharge from RICU, or even discharge from the hospital. The general condition of the patients in the two groups was comparable (Table 1).

Fourteen patients in the HDU group received IMV (mean invasive ventilation time: 176 ± 160 h) as against 24 in the normal group (mean invasive ventilation time: 206 ± 179 h). Most patients received sequential NIV or HFNO after IMV. In these patients, NIV or HFNO time was defined as “sequential noninvasive ventilation period”. The sequential noninvasive ventilation period in the HDU group (135 ± 82 h) was significantly shorter less than that in the normal group (274 ± 182 h; P < 0.05) (Table 2).

**Table 2:** Comparison of ventilation period, ICU complications, and prognosis.

	HDU group, n = 40	Normal group, n = 47	t/X^2^/M-W	P
Invasive ventilation (n, %)	14 (35.0)	24 (51.1)	2.267	0.132
Invasive ventilation period (h)	176 ± 160	206 ± 179	− 0.524	0.603
Sequential noninvasive ventilation period (h)	135 ± 82	274 ± 182	− 2.594	0.017
Noninvasive ventilation (n, %)	26 (65.0)	23 (48.9)	2.267	0.132
Noninvasive ventilation period (h)	125 (72, 187)	158 (82, 341)	220.5	0.334
ICU syndrome (n, %)	0	1 (2.1)	0.861	1.000
Culture yield (including colonized bacteria and pathogenic bacteria) (n, %)	17 (42.5)	25 (53.2)	0.999	0.320
Returning ICU within 48 h (n, %)	2 (5.0)	3 (6.4)	0.076	1.000
Mortality (n, %)	2 (5.0)	4 (8.5)	0.415	0.683
Length of stay in RICU	5.25 (3.5, 8.0)	8.5 (5.5, 15.0)	619.0	0.006
Length of stay in hospital	13.0 (8.5, 16.5)	25.5 (12.0, 34.0)	544.0	0.001
Expenditure (CNY)	55,453 (38,271, 90,302)	99,492 (54,538, 201,853)	533.0	0.001

Data presented as frequency (%), mean ± standard deviation or as median (interquartile range).

1 USD = 6.455 CNY; USD, USA dollar; CNY, China Yuan.

*RICU* respiratory intensive care unit, *ICU* intensive care unit, *HDU* high dependency unit.

While some patients never required IMV but NIV or HFNO. NIV or HFNO time was defined “noninvasive ventilation period” in this sort of patients. Twenty-six patients in the HDU group received NIV (median ventilation period: 125 (72, 187) h) as against 23 in the normal group (median ventilation period: 158 (82, 341) h; P > 0.05) (Table 2).

The number of deaths in the HDU and normal group were 2 (5.0%) and 4 (8.5%), respectively (P > 0.05). There was no significant between-group difference with respect to the incidence of ICU syndrome, culture yield, or the rate of return to ICU within 48 h (Table 2).

**Figure 2:**
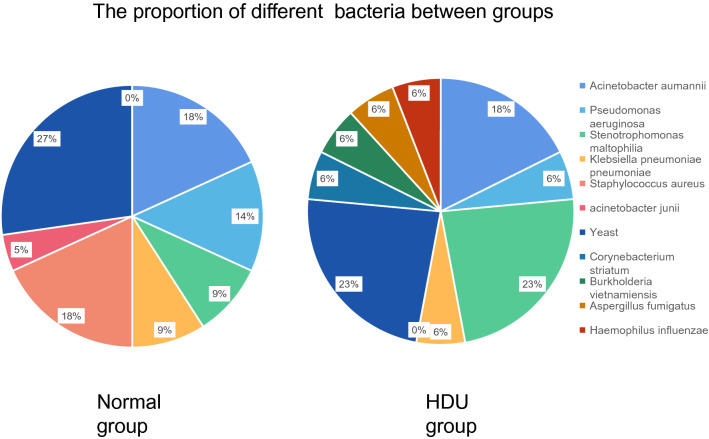
The proportion of different bacteria between groups.

**Figure 3:**
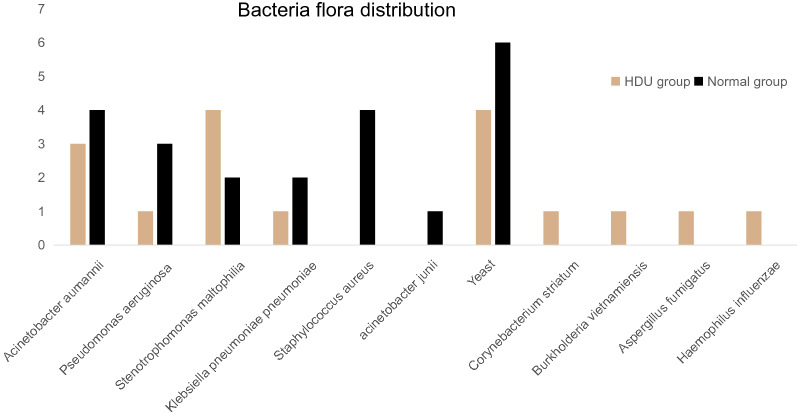
Bacteria flora distribution between groups.

However, it is worth mentioning that the distribution of bacterial flora was significantly different between the two groups. The main organisms in the HDU group were Stenotrophomonas maltophilia and yeast compared with Staphylococcus aureus, Acinetobacter aumannii, and yeast in the normal group (Table 3, Figs. 2, 3).

**Table 3:** Distribution of bacteria flora in the two groups.

Bacteria (n, %)	HDU group (n = 17)	Normal group (n = 25)	P
Acinetobacter aumannii	3 (17.6)	4 (16.0)	1.000
Pseudomonas aeruginosa	1 (5.9)	3 (12.0)	0.635
Stenotrophomonas maltophilia	4 (23.5)	2 (8.0)	0.202
Klebsiella pneumoniae pneumoniae	1 (5.9)	2 (8.0)	1.000
Staphylococcus aureus	0	4 (16.0)	0.134
acinetobacter junii	0	1 (4.0)	1.000
Yeast	4 (23.5)	6 (24.0)	1.000
Corynebacterium striatum	1 (5.9)	0	0.405
Burkholderia vietnamiensis	1 (5.9)	0	0.405
Aspergillus fumigatus	1 (5.9)	0	0.405
Haemophilus influenzae	1 (5.9)	0	0.405

*HDU* high dependency unit.

On the other aspects, the median length of stay in the hospital [13.0 (8.5, 16.5) days] and RICU [5.25 (3.5, 8.0) days] in the HDU group were shorter than those in the normal group [25.5 (12.0, 34.0) days and 8.5 (5.5, 15.0) days, respectively]. Moreover, the median expenditure incurred by patients in the HDU group [CNY 55453 (38271, 90302)] was significantly lesser than that in the normal group [CNY 99492 (54538, 201853) (P < 0.05)] (Table 2).

## Discussion

The incidence of SCAP has shown an increasing trend over the last decades and it is reported as one of the most common causes of sepsis in hospitalized patients^12^. SCAP is not only associated with high short-term and long-term mortality rates but also imposes a huge burden in clinical settings^13^. Hence, improving the management strategy for SCAP is a key imperative. SCAP usually necessitates ICU admission, mainly because of the need for mechanical ventilation or vasoactive agents. However, ICU admission can cause some complications, such as secondary infection, malnutrition, and ICU syndrome, which can lead to increased expenditure and prolonged length of stay (LOS) in ICU.

HDU, a setting outside of ICU but with critical care, provides a new pattern to manage SCAP patients. As a bridge between the ICU and the normal unit, HDU can allow provision of both close supervision and family company^14^.

In our study, we compared the two management patterns of RICU-HDU-NU and RICU-NU in SCAP patients. We analyzed the clinical data of 87 SCAP patients including 40 in the HDU group and 47 in the normal group.

We found that patients in both two groups showed increase in oxygenation index and reduction in APACHE II and SOFA scores through treatment. There was no significant between-group difference with respect to oxygenation index, APACHE II score and SOFA score at the time of admission or discharge from RICU or the rate of return to ICU within 48 h. This implied a comparable therapy effect in the two groups. Instead, patients in the HDU group had less sequential noninvasive ventilation period, shorter LOS in hospital and RICU, and lower expenditure. As expected, the RICU-HDU-NU pattern decreased the LOS and expenditure, but it did not decrease the therapy effect, which illustrates that this pattern can be developed for management of SCAP.

Hukins et al. presented real-life data on the outcomes of HDU management of patients with respiratory failure at a tertiary care hospital in Australia. They found that use of NIV for treatment of hypoxaemic respiratory failure in HDU is effective in most patients^15^.

The following reasons may explain the comparable outcomes in the two groups: (1) The same medical and nursing team was responsible for both groups to maintain the continuity in therapy, even though there were relatively lesser nurses in the HDU. With the stabilization of the patient’s condition and help of family, a nurse-patient ratio of 1:4 is acceptable. (2) With respect to bacteria culture yield, Stenotrophomonas maltophilia and Yeast were the most commonly isolated organisms in the HDU group, which are not among the most common causes of hospital-acquired infection and less likely to be antibiotic-resistant. Nevertheless, Staphylococcus aureus and Acinetobacter aumannii, which are among the top 5 bacteria causing nosocomial infection according to 2021 CHINET data, were the main bacteria in the normal group, which are more likely to develop drug resistance. Thus, maybe HDU has a lower risk of nosocomial infection and drug resistance. (3) Transferring the patient out of ICU as soon as possible decreases the LOS in ICU and minimizes the risk of secondary infection. (4) In addition, the presence of family members helps decrease the risk of ICU syndrome and promote patients’ rehabilitation physiotherapy and illness recovery. As the results showed, there was shorter sequential noninvasive ventilation period in HDU group than that in normal group. This may be related to the shorter period of LOS in hospital. Decreased LOS is liable to reduce the expenditure.

In our study, although there was no significant difference with respect to the distribution of causative organisms between the two groups, we found that most bacteria in HDU group seemed unfamiliar to normal ICU and may be less likely to be drug-resistant. A larger study in future may show significant difference in bacteria distribution. Moreover, inclusion of drug sensitivity test for all pathogens in future may help characterize the microbial profile in the two groups.

The mortality rate of patients with SCAP in the HDU and normal groups were 5% and 8.5%, respectively, which is lower than that in previous studies^16,17^. This was because we only analyzed the patients who had been transferred out of RICU, regardless of whether they were readmitted to RICU again or not. There were 20 patients who could not be extubated and died in RICU. Inclusion of these cases would increase the mortality rate to approximately 24.3%, which is closer to that reported in the study by Waldens et al.^17^.

Our study is of much clinical significance. We highlight a new strategy for management of SCAP which helped achieve good results. The RICU-HDU-NU pattern is worth developing for the treatment of SCAP patients. Moreover, establishing HDU can help ease the bed burden of RICU and nurses and improve the bed rotation rate, an assessment indicator in hospitals. Third, the company of family members in the HDU improves the satisfaction of patients and family members.

Owing to the increasing burden of severe pneumonia due to the COVID-19 pandemic, many countries have started to develop HDU. However, the concept of HDU is not widely developed in China, and it is mostly designed for surgical patients^18^. The development of internal medicine HDU lags behind that of surgical HDU^19–21^. Our research may provide a new idea to develop internal medicine HDU in each specialty.

Some limitations of our study should be acknowledged. This was a retrospective, single-center study with a small sample size. Multicenter prospective studies are required to provide more definitive evidence of the benefits of HDU.

## Data Availability

The data used to support the findings of this study are available from the corresponding author upon request.
